# Machine learning to predict late respiratory support in preterm infants: a retrospective cohort study

**DOI:** 10.1038/s41598-023-29708-4

**Published:** 2023-02-17

**Authors:** Tsung-Yu Wu, Wei-Ting Lin, Yen-Ju Chen, Yu-Shan Chang, Chyi-Her Lin, Yuh-Jyh Lin

**Affiliations:** 1 Department of Pediatrics, Ditmanson Medical Foundation Chia-Yi Christian Hospital, Chiayi, Taiwan; 2 Division of Neonatology, Department of Pediatrics, National Cheng Kung University Hospital, College of Medicine, National Cheng Kung University, No.138, Sheng Li Rd., North Dist., Tainan, 704 Taiwan; 3 Department of Pediatrics, E-Da Hospital, I-Shou University, Kaohsiung, Taiwan; 4 School of Medicine for International Students, College of Medicine, I-Shou University, Kaohsiung, Taiwan

**Keywords:** Respiratory tract diseases, Paediatrics, Outcomes research, Paediatric research

## Abstract

Bronchopulmonary dysplasia (BPD) has been a critical morbidity in preterm infants. To improve our definition and prediction of BPD is challenging yet indispensable. We aimed to apply machine learning (ML) to investigate effective models by using the recently-proposed and data-driven definition to predict late respiratory support modalities at 36 weeks’ post menstrual age (PMA). We collected data on very-low-birth-weight infants born between 2016 and 2019 from the Taiwan Neonatal Network database. Twenty-four attributes associated with their early life and seven ML algorithms were used in our analysis. The target outcomes were overall mortality, death before 36 weeks’ PMA, and severity of BPD under the new definition, which served as a proxy for respiratory support modalities. Of the 4103 infants initially considered, 3200 were deemed eligible. The logistic regression algorithm yielded the highest area under the receiver operating characteristic curve (AUROC). After attribute selection, the AUROC of the simplified models remain favorable (e.g., 0.801 when predicting no BPD, 0.850 when predicting grade 3 BPD or death before 36 weeks’ PMA, and 0.881 when predicting overall mortality). By using ML, we developed models to predict late respiratory support. Estimators were developed for clinical application after being simplified through attribute selection.

## Introduction

Despite recent progress in neonatal care, bronchopulmonary dysplasia (BPD) remains a critical morbidity of preterm infants^1–3^. BPD, first described by Northway in 1967, is a lung disease observed following recovery from respiratory distress syndrome and from aggressive mechanical ventilation with high concentration of oxygen^4^.

BPD has a critical impact on subsequent mortality and morbidity, which places a heavy burden on families and society^5–7^. Infants with BPD are at higher risk of multiple rehospitalization and longer duration of hospitalization^8^. Long-term follow-up of BPD survivors has revealed poorer pulmonary health and abnormal lung function tests, even into late adolescence^9,10^. Moreover, BPD has a strong association with neurodevelopmental impairments or survival with disability in long-term follow-up^5,6^.

The definition of BPD has evolved considerably over decades. It was first characterized by Tooley as the oxygen dependence (e.g., fraction of inspired oxygen requirement [FiO2] > 21%) at the 30th day after birth^11^. Shennan suggested that the requirement for additional oxygen at a postmenstrual age (PMA) of 36 weeks could better predict adverse pulmonary health than previous criteria^12^. In 2001, the National Institute of Child Health and Human Development held a consensus conference at which a definition of BPD with distinct severities was proposed (Table 1)^13^. However, refining the definition has been an ongoing challenge. Defining BPD differently can alter the disease incidence, which ranges from 6 to 57%^14^. Recent changes in respiratory management, such as high-flow nasal cannula, limit the applicability of previous definitions^15^. Jensen therefore conducted a study to develop an evidence-based, or data-driven, definition of BPD; they concluded that the mode of respiratory support administered at 36 weeks’ PMA, regardless of supplemental oxygen, can best predict early childhood morbidity. (Table 1)^16^.

**Table 1:** Definitions of bronchopulmonary dysplasia.

Definition	NICHD		Jensen	
Time Point of Assessment	At 36 weeks’ PMA or at discharge, whichever comes first		At 36 weeks’ PMA or at discharge, whichever comes first	
Consideration of oxygen supplement	Treatment with oxygen > 21% for at least 28 days plus			
Severity or grading of BPD	Mild BPD	Breathing room air	No BPD	Room air, no support
	Moderate BPD	Need for oxygen < 30%	Grade 1 BPD	NC ≤ 2L/min “low flow”
	Severe BPD	Need for oxygen ≥ 30% and/or positive pressure (PPV or NCPAP)	Grade 2 BPD	NC > 2L/min “high flow,” or NCPAP or NIPPV
			Grade 3 BPD	Invasive PPV

*BPD* Bronchopulmonary dysplasia; *GA* Gestational age; *NC* Nasal cannula; *NCPAP* Nasal continuous positive airway pressure; *NIPPV* Nasal intermittent positive pressure ventilation; *NICHD* National Institute of Child Health and Human Development; *PMA* Postmenstrual age; *PPV* Positive-pressure ventilation.

Machine learning (ML) technique is a tool that involves the use of algorithms to make sense of a tremendous amount of structured (e.g., numbers) or unstructured (e.g., images) data^17,18^. ML differs from the traditional approaches in that it involves learning from examples themselves instead of being designed to function on the basis of static rules alone. A model can be developed and trained to predict certain patterns or outcomes by using a large volume of data^19,20^. Recently, the technique has been increasingly applied in medical fields.

The ability to predict the outcomes of premature infants from their early life onward can aid in treatment planning, family counseling, and even individualized management.

The aim of this study, therefore, was to use ML technique to establish optimal models for predicting mortality and respiratory support modalities in very-low-birth-weight preterm infants at 36 weeks’ PMA.

## Materials and methods

### Materials

We retrospectively collected cohort data on very-low-birth-weight infants born between 2016 and 2019 from the Taiwan Neonatal Network (TNN) database, which was established in 2016 and was designed to store nationwide clinical information for premature neonates born in Taiwan. Between 2016 and 2019, 24 hospitals, including the majority of secondary and tertiary neonatal intensive care units in Taiwan, joined the TNN.

The inclusion criteria were a gestational age (GA) of 22 weeks, 0 days to 31 weeks, 6 days or a birth weight (BW) of 401–1500 g. The exclusion criteria were having died within 12 h after birth or admission or having received a diagnosis of congenital anomaly. The definition of congenital anomaly encompassed chromosomal anomalies, skeletal dysplasia, inborn error of metabolism, lethal or life-threatening anomalies in the cardiovascular, gastro-intestinal, genito-urinary, or pulmonary systems, and other lethal or life-threatening anomalies.

We collected data on 24 early-life characteristics as attributes: antenatal steroid use, magnesium sulfate use, chorioamnionitis, maternal hypertension, Caesarean section, multiple birth, the first-minute Apgar score, the fifth-minute Apgar score, noninitiated initial neonatal resuscitation, initiated neonatal resuscitation (including the use of oxygen supply, face-mask ventilation, intubation, epinephrine administration, chest compression or continuous positive airway pressure), the grading of initial neonatal resuscitation, sex, GA, BW, whether small for gestational age^21^, birth place, early onset sepsis (a positive blood and/or cerebral spinal fluid culture within 3 days of birth), respiratory distress syndrome, and the use of surfactants. The time point to predict BPD in our study was 72 h after birth. The GA was presented only in completed weeks and the BW was presented only in 100 g bins. The z scores therefore could not be calculated.

We defined the grading of initial neonatal resuscitation to classify the disease severity during neonatal resuscitation. We hierarchically defined that noninitiated initial neonatal resuscitation, the use of oxygen supply, continuous positive airway pressure, face-mask ventilation, intubation, epinephrine administration, and chest compression to be mild to most severe. Respiratory distress syndrome was defined as:

1. Within the first 24 h of life, a chest radiograph consistent with the characteristics of respiratory distress syndrome, such as reticulogranular appearance to lung fields, air-bronchograms, with or without decreased lung volumes.
2. Plus at least one of the criteria below: partial pressure of oxygen < 50 mmHg in room air, central cyanosis in room air, a requirement for supplemental oxygen to maintain partial pressure of oxygen > 50 mmHg, and/or a requirement for supplemental oxygen to maintain a pulse oximeter saturation > 85%.

This study has been approved by the National Cheng Kung University Hospital Institutional Review Board (A-ER-109–181). The need of informed consent was waived by the National Cheng Kung University Hospital Institutional Review Board due to the fact that data were anonymized and de-identified. All methods were performed in accordance with the relevant guidelines and regulations.

### Target outcomes

**Figure 1:**
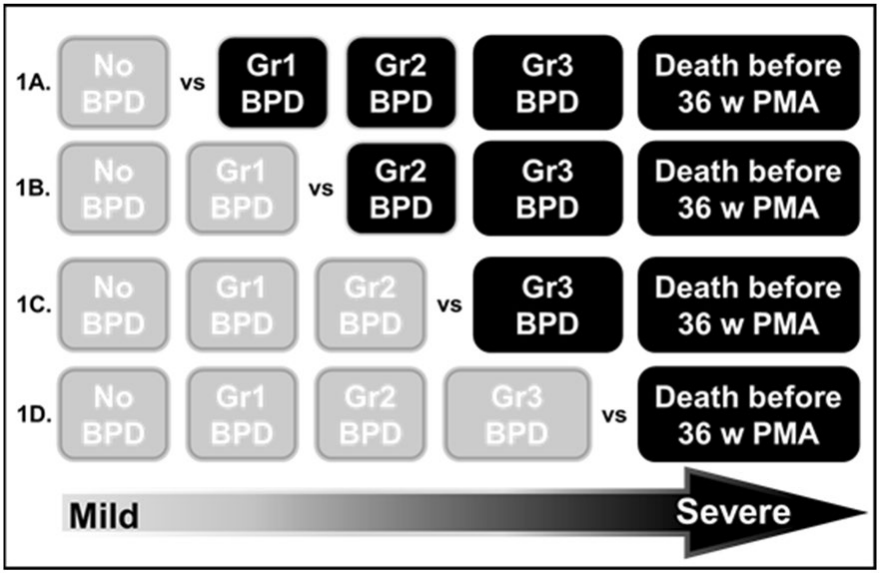
Binary classifications of various target outcomes. 1(**A**): No BPD versus other conditions; 1(**B**): Grade 1 BPD or no BPD versus other conditions; 1(**C**): Death before 36 weeks’ PMA or Grade 3 BPD versus other conditions; 1(**D**): Death before 36 weeks’ PMA versus other conditions; *BPD* Bronchopulmonary dysplasia; *Gr* Grade; *PMA* Postmenstrual age.

Our primary outcomes were the needs of late respiratory support modalities at 36 weeks’ PMA. According to the definition of BPD proposed by Jensen^16^, the respiratory support at 36 weeks’ PMA were categorized as the grading of BPD (Table 1). Those who died before 36 weeks’ PMA were grouped together because they could not be assigned any BPD grade. For each predictive model, the following binary target outcomes were determined: (Fig. 1).

1. No BPD versus other conditions.
2. Grade 1 BPD or no BPD versus other conditions.
3. Death before 36 weeks’ PMA or Grade 3 BPD versus other conditions.
4. Death before 36 weeks’ PMA versus other conditions.

In addition, the secondary outcome in this study was overall mortality versus other conditions.

### Statistics analysis

#### Model development and comparison

**Figure 2:**
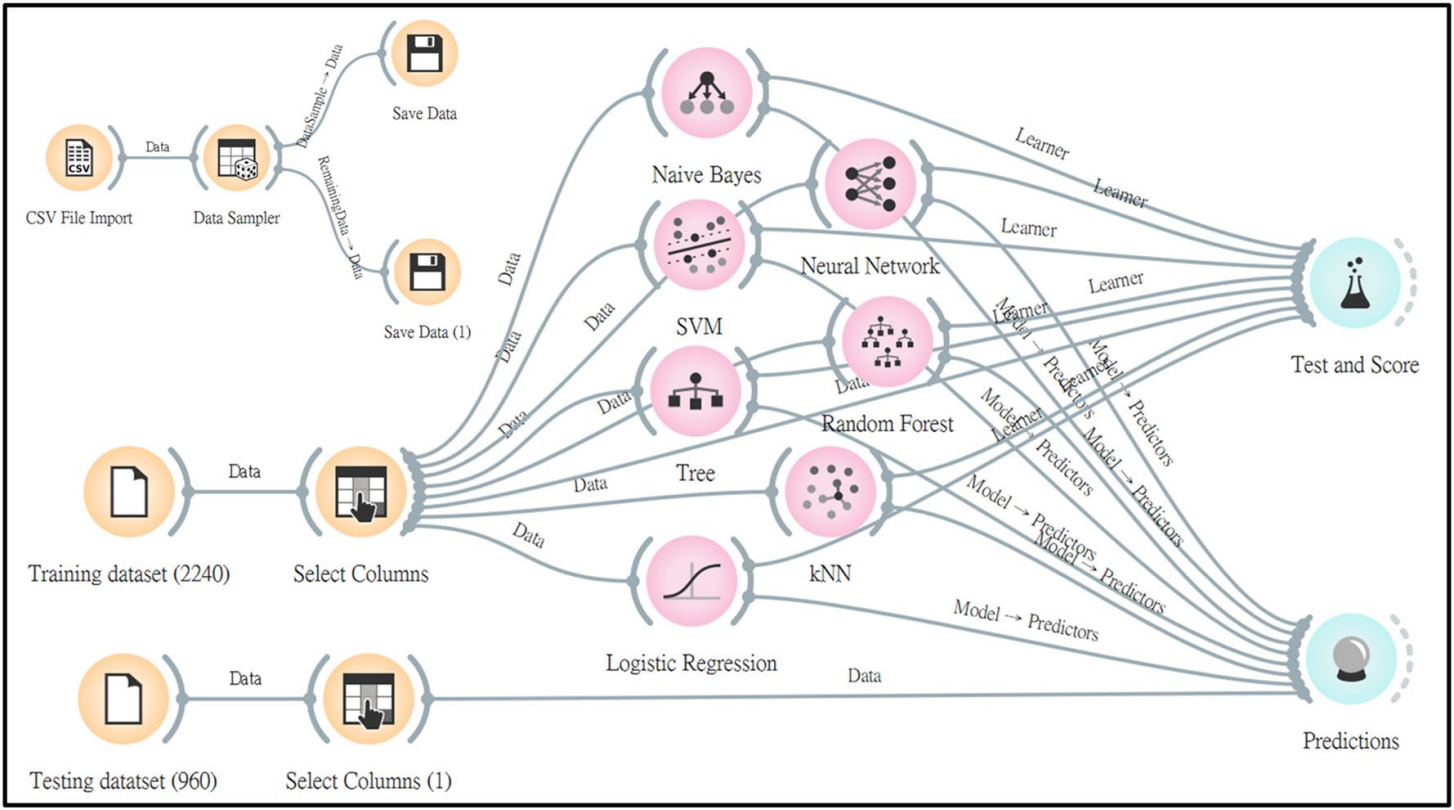
Workflow for Orange software, Version 3.27.1. *SVM* Support vector machine; *Tree* Classification tree; *kNN* k-nearest neighbor.

We utilized Orange software, (version 3.27.1; Bioinformatics Lab, Ljubljana, Slovenia) to analyze our data^22^. First, we separated our data randomly into two subsets: the training data set, which consisted of 70% of the cohort, and the testing data set, which consisted of the remaining 30%. The workflow is illustrated in Fig. 2. We loaded our training and testing data sets and then selected different target outcomes. Distinct algorithms, such as classification tree, k nearest neighbor, logistic regression, naïve Bayes, neural network, random forest, and support vector machine, were used for model building. The models were constructed with the training data set and were evaluated with tenfold cross validation. The remaining 30% of the cohort, namely the testing data set, were used for internal validation. The area under the receiver operating characteristic curve (AUROC) of each model was calculated to evaluate model performance. Attribute selection and equation development for outcome estimation were then applied to the algorithm with the highest AUROC.

#### Attribute selection and simplified models

For clinical applications, we utilized Weka software (version 3.8.4; Waikato Environment for Knowledge Analysis, Hamilton, New Zealand) for attribute selection^23^. After applying the CfsSubsetEval function with BestFirst search method in Weka, some attributes from the original 24 attributes for distinct target outcomes were selected by using the training data set. The attribute selection was performed entirely software-based, or data-driven, and not based on biological plausibility or any clinical considerations. In Orange, we applied the attributes selected by Weka along with the optimal algorithm (i.e., that with the highest AUROC value) to develop simplified models for various outcomes. The 70–30% training–testing data set split and tenfold cross validation were also applied. The AUROCs of the simplified models were examined and compared with the previous complex models before attribute selection.

#### Equation development

We used Orange to calculate the intercept and each coefficient of each selected attribute for the various outcomes. The equations were developed thereafter. Finally, we developed estimators to predict the probability of the various target outcomes.

## Results

### Study population and patient characteristics

**Figure 3:**
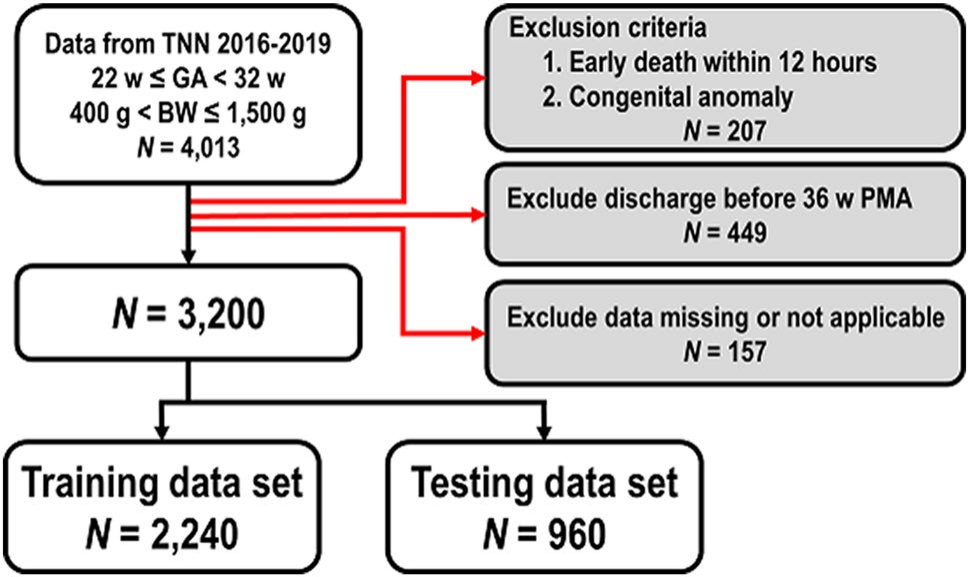
Patient selection flowchart. *BW* Birth weight; *GA* Gestational age; *PMA* Postmenstrual age; *TNN* Taiwan neonatal network.

This study enrolled 4013 infants. We excluded 207 infants because they had died within 12 h after birth or after admission, or because they had congenital anomalies. Another 449 infants were excluded because they were discharged before 36 weeks’ PMA and the applied respiratory support modalities could not be determined. Furthermore, 157 infants were excluded due to missing or inapplicable data. In total, 3200 infants were eligible for the final analysis. The cohort was then split randomly into 2 data sets by applying randomization in Orange. The training data set (*N* = 2240) consisted of 70% of all data and was applied in models development. The remaining 30% were assigned to the testing data set (*N* = 960), which was applied for internal validation of each model (Fig. 3).

The characteristics of the enrolled infants and of the two data sets are detailed in Table 2. In our cohort, the mean GA was 27.47 ± 2.35 weeks, and the mean BW was 928 ± 280 g. The only significant difference between the training and testing data sets was in the proportion of small for GA (Training: 30% vs. Testing: 32%, *p* < 0.05).

**Table 2:** Patient characteristics.

Characteristics	All	Training data set	Testing data set	*p* value
Patient number, n (%)	3200 (100%)	2240 (70%)	960 (30%)	
GA, week, mean ± SD	27.47 ± 2.35	27.48 ± 2.35	27.44 ± 2.35	0.613
BW, per 100 g, mean ± SD	9.28 ± 2.80	9.27 ± 2.80	9.29 ± 2.79	0.832
SGA*, n (%)	1014 (32%)	725 (30%)	289 (32%)	0.029**
Sex, female, n (%)	1496 (47%)	1,050 (47%)	446 (46%)	0.829
Outborn, n (%)	203 (6%)	132 (6%)	71 (7%)	0.110
Maternal history				
Antenatal corticosteroid, n (%)	2781 (87%)	1950 (87%)	831 (87%)	0.706
Antenatal MgSO4, n (%)	1926 (60%)	1349 (60%)	577 (60%)	0.950
Chorioamnionitis, n (%)	580 (18%)	397 (18%)	183 (19%)	0.367
Maternal hypertension, n (%)	747 (23%)	521 (23%)	226 (24%)	0.862
Caesarean section, n (%)	2,239 (70%)	1580 (71%)	659 (69%)	0.285
Multiple births, n (%)	936 (29%)	658 (29%)	278 (29%)	0.812

*BW* Birth weight; *GA* Gestational age; *SD* Standard deviation; *SGA* Small for gestational age.

*Reference: Hsieh^21^.

**p < 0.05.

### Model development and comparison

The AUROC values of the prediction models developed using the various algorithms are listed in Table 3. For each algorithm, the differences in AUROC values between the training and testing data sets were small, indicating that overfitting was avoided. Of the seven distinct algorithms, logistic regression registered the highest AUROC values in predicting the various target outcomes. In the testing data set, the AUROC values of logistic regression were 0.812 when predicting no BPD, 0.769 when predicting no BPD or grade 1 BPD, 0.854 when predicting grade 3 BPD or death before 36 weeks’ PMA, 0.884 when predicting death before 36 weeks’ PMA, 0.884 when predicting overall mortality. Therefore, we used logistic regression in attribute selection and the development of simplified prediction models.

**Table 3:** AUROC values of each model developed using various algorithms and training and testing data sets, and AUROC values of logistic regression after attribute selection using training and testing sets (Italic Data).

Algorithms	Outcomes	No BPD	No BPD or Gr1 BPD	Gr3 BPD or death before 36w PMA	Death before 36w PMA	Overall mortality
kNN	Training data set	0.735	0.720	0.762	0.744	0.746
	Testing data set	0.753	0.708	0.789	0.805	0.792
Logistic regression	Training data set	0.803	0.777	0.811	0.833	0.831
	Testing data set	0.812	0.769	0.854	0.884	0.884
Naïve bayes	Training data set	0.783	0.757	0.789	0.819	0.817
	Testing data set	0.781	0.736	0.841	0.877	0.879
Neural network	Training data set	0.761	0.735	0.779	0.783	0.785
	Testing data set	0.766	0.721	0.786	0.814	0.818
Random forest	Training data set	0.765	0.747	0.780	0.784	0.780
	Testing data set	0.765	0.733	0.819	0.857	0.845
SVM	Training data set	0.645	0.647	0.631	0.659	0.639
	Testing data set	0.664	0.623	0.670	0.804	0.708
Classification tree	Training data set	0.632	0.645	0.583	0.587	0.560
	Testing data set	0.682	0.642	0.646	0.608	0.702
*After attribute selection*						
*Logistic regression*	*Training data set*	*0.802*	*0.776*	*0.811*	*0.835*	*0.833*
	*Testing data set*	*0.801*	*0.763*	*0.850*	*0.881*	*0.881*

*AUROC* Area under the receiver operating characteristic curve; *BPD* Bronchopulmonary dysplasia; *kNN* k-nearest neighbors; *Gr* Grade; *PMA* Postmenstrual age; *SVM* Support vector machine.

### Attribute selection and simplified models

Among all 24 attributes, 5–7 attributes were selected for various target outcomes by using Weka, and the results were indicated in Table 4. Five attributes were selected for all target outcomes: BW, GA, intubation during initial neonatal resuscitation, early sepsis, and the administration of surfactant.

**Table 4:** Attributes selected for various target outcomes by weka.

Target outcomes	Selected attributes
No BPD	Birth weight, gestational age, intubation*, early sepsis**, surfactant***
No BPD or Gr1 BPD	Birth weight, gestational age, intubation*, early sepsis**, Respiratory distress syndrome, surfactant***
Gr3 BPD or death before 36 w PMA	Birth weight, gestational age, sex, intubation*, early sepsis**, surfactant***
Death before 36 w PMA	Birth weight, gestational age, the first-minute apgar score, intubation*, epinephrine*, early sepsis**, surfactant***
Overall mortality	Birth weight, gestational age, the fifth-minute apgar score, intubation*, epinephrine*, early sepsis**, surfactant***

*BPD* Bronchopulmonary dysplasia; *Gr* Grade; *PMA* Postmenstrual age.

*Had received intubation or epinephrine administration during neonatal resuscitation in the delivery or operation room.

**A positive culture report from a blood sample and/or a cerebrospinal fluid sample obtained on day 1, 2, or 3 of life.

***Exogenous surfactant administrated at any time and through any pathway.

Using these selected attributes, we used Orange and logistic regression algorithm to construct simplified prediction models. The AUROC values for predicting the various outcomes after attribute selection are listed in Table 3 (Italic data). The differences in AUROC values between the training and testing data sets were still small. Compared with the previous complex models, the simplified models had similar AUROC values. The AUROC values in the testing data set of the simplified models were 0.801 when predicting no BPD, 0.763 when predicting no BPD or grade 1 BPD, 0.850 when predicting grade 3 BPD or death before 36 weeks’ PMA, 0.881 when predicting death before 36 weeks’ PMA, 0.881 when predicting overall mortality. Therefore, the simplified models maintained favorable performance in predicting the various target outcomes. Finally, to enhance applications for clinical practice, the logistic regression–based simplified models were used in equation development.

### Equation development

The equation of the logistic regression algorithm can be written as Eq. (1).

1 $$\mathrm{P}=1-\frac{1}{1+\mathrm{exp}[-\left(W0+W1X1+W2X2+W3X3+W4X4+W5X5+\dots \right)]}$$

**Figure 4:**
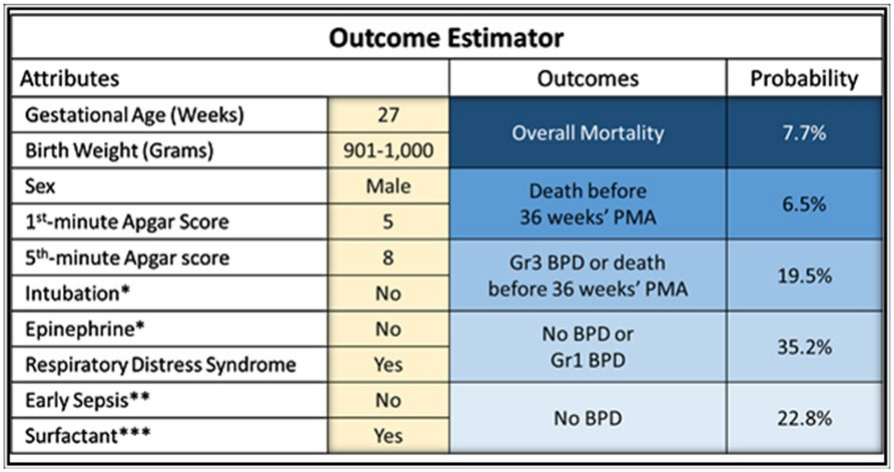
Outcome estimator. *BPD* Bronchopulmonary dysplasia; *Gr* Grade; *PMA* Postmenstrual age. *Had received intubation, epinephrine administration, or chest compression during neonatal resuscitation in the delivery or operation room. **A positive culture report from a blood sample and/or a cerebrospinal fluid sample obtained on day 1, 2, or 3 of life. ***Exogenous surfactant administered at any time or through any pathway.

We used Orange to calculate the intercept and coefficient of each selected attribute for the prediction models constructed using logistic regression. The results are listed in Table 5. An equation was developed for each target outcomes, and finally, outcome estimators for clinical applications were established using Microsoft Excel 2016 (Fig. 4) (Supplementary information).

**Table 5:** Intercept and coefficient values of the attributes in various models developed using logistic regression.

Target outcomes	No BPD	No BPD or Gr1 BPD	Gr3 BPD or death before 36w PMA	Death before 36w PMA	Overall mortality
Intercept	− 9.05217	− 4.50364	2.06853	2.03494	2.54808
GA	0.248647	0.116522	− 0.0750603	− 0.0930285	− 0.0777832
BW	0.18391	0.223937	− 0.306912	− 0.291437	− 0.304774
Sex (male)			0.425695		
1st-min Apgar score				− 0.0680075	
5th-min Apgar score					− 0.128508
Intubation*	− 0.604876	− 0.28599	0.490307	0.655439	0.504843
Epinephrine*				0.716202	0.447399
RDS		− 0.983329			
Early sepsis**	− 0.819743	− 0.580436	0.679287	1.11624	0.904317
Surfactant***	− 0.53518	− 0.283135	0.876138	0.767558	0.833243

*BPD* Bronchopulmonary dysplasia; *BW* Birth weight; *GA* Gestational age; *Gr* Grade; *PMA* Postmenstrual age; *RDS* Respiratory distress syndrome.

*Had received intubation or epinephrine administration during neonatal resuscitation in the delivery or operation room.

**A positive culture report from a blood sample and/or a cerebrospinal fluid sample obtained on day 1, 2, or 3 of life.

***Exogenous surfactant administrated at any time and through any pathway.

For example, assume a premature male infant is born at a GA of 27 complete weeks with a BW of 901–1000 g. His first-minute and fifth-minute Apgar scores are 5 and 8, respectively. He does not receive intubation or epinephrine administration during initial neonatal resuscitation. A diagnosis of respiratory distress syndrome is made and he receives exogenous surfactant replacement after birth. He does not have early sepsis. In summary, this boy’s likelihood of overall mortality is 7.7%, death before 36 weeks’ PMA is 6.5%, grade 3 BPD or death before 36 weeks’ PMA is 19.5%, no BPD or grade 1 BPD is 35.2%, and, no BPD is 22.8% (Fig. 4).

## Discussion

In this study, we developed models to predict the probability of respiratory support at 36 weeks’ PMA from preterm infants’ early-life characteristics. Among the various algorithms, the models developed using logistic regression exhibited the optimal performance. Each estimator was established for clinical applications after being simplified through attribute selection.

With medical improvements, increasing numbers of premature infants are surviving, but the incidence of BPD remains similar or may even have increased^1–3^. Accurate prediction of BPD may provide opportunity for prevention and benefit not only patients but also clinicians, parents and relevant researchers. Over the past decades, several predictive models or scoring systems have been proposed but each had its own limitations^24^.

Ryan used logistic regression to develop prediction models that were based on infants’ early-life characteristics, and Romagnoli created a scoring system for predicting BPD^25,26^. However, neither of them considered death before the diagnosis of BPD as a competing outcome. Noack and Yuksel both applied chest radiological findings and developed scoring systems to predict BPD^27,28^. Yet, the interpretation of chest radiographs was excessively subjective and lacked generalizability. Moreover, the definitions of BPD used by these four studies were outdated and therefore unsatisfactory for use in contemporary medicine.

An estimator for the likelihood of death or BPD of various severities was developed on the basis of data from the largest relevant multicenter study conducted by Laughon^29^. In that study, six risk factors were selected to construct a BPD estimator, including GA, BW, race and ethnicity, sex, respiratory support and FiO2, and the contribution of risk factors was concluded to be subject to change depending on postnatal age when predicting BPD. However, the study did not consider high-flow nasal cannula, which is currently a common ventilator support used for neonates^30^.

Katherine suggested that early cumulative supplemental oxygen may be a predictor of BPD or death, with cumulative supplemental oxygen at 14 days having the optimal predictive accuracy^31^. However, this study was conducted with a restricted and high-risk cohort. The application of the FiO2 or the peak inspiratory pressure as variables often results in a lack of generalizability because distinct units may have different policies regarding target saturation or blood gas data. To accurately record the cumulative FiO2 or daily peak inspiratory pressure may be time-consuming and cumbersome.

Gursoy developed a clinical scoring system to predict BPD at as early as 72 h after birth^32^. They defined and categorized the severity of BPD using the National Institute of Child Health and Human Development criteria and developed a scoring system by using clinical parameters and achieved good performance (AUROC = 0.930), even in the validation group (AUROC = 0.903). However, the study cohort was relatively small, and death before 28 days of life was not considered to be a competing outcome.

Our study has several notable strengths. First, this was a nationwide population-based cohort study in Taiwan. Data from over 80% of total very-low-birth-weight infants in Taiwan were uploaded annually. Second, our study adopted the new definition proposed by Jensen in 2019 for BPD diagnosis and categorization^16^. Although the diagnostic criteria for BPD are continually evolving, the criteria of Jensen are based on data science instead of mere expert opinion and have been demonstrated to be more informative when predicting early childhood morbidities. Moreover, these criteria also involve consideration of contemporary respiratory care, such as high-flow nasal cannula, and circumvent the need to calculate supplemental oxygen use, which is practically challenging due to differing treatment policies on respiratory care between hospitals. We therefore employed these criteria to grade BPD and to serve as a proxy for respiratory support at a PMA of 36 weeks. Third, we selected distinct severities of BPD in binary classifications as our target outcomes; for instance, grade 3 BPD or death before 36 weeks’ PMA versus other conditions (i.e., no BPD, grade 1 BPD or grade 2 BPD). Such binary classification can be easily interpreted and understood. In addition, competing outcome were not omitted. Finally, ML techniques were applied to analyze our data. The noteworthy strength of ML is its ability to yield data-driven findings by using a large volume of data after being trained again and again. Such an approach could teach an algorithm, including logistic regression, to build a model with high performance. In our study, it was the logistic regression algorithm that demonstrated the most promising performance, with AUROCs of approximately 0.8. For superior clinical applications, attribute selection was performed automatically by ML technique by Weka. The AUROCs remained similar. We demonstrated that even after attribute selection, the simplified models continued to function just as favorably. However, overfitting is a common drawback when using ML. Our study used a completely unseen dataset, namely, the testing dataset, for validation and demonstrated that overfitting was avoided. By using our estimator, we were able to predict a preterm infant’s outcome from early-life characteristics, and subsequent preventive or therapeutic treatment strategies could be planned.

There are several different ways to select independent variables in a logistic regression model, such as domain knowledge (expert knowledge), correlation, statistical tests, stepwise selection, and others. Our study utilized the CfsSubsetEval function, which is one of the correlation-based feature selections, in Weka to perform attribute selection. The technique can identify and compute attributes that are correlated or predictive of the class but uncorrelated with one another. Different from performing attributes selection in a logistic regression model, the CfsSubsetEval attribute selection in Weka is performed before building a model with the algorithm. The CfsSubsetEval attribute selection is based on the principle of “A good feature subset is one that contains features highly correlated with (predictive of) the class, yet uncorrelated with (not predictive of) each other”^33^. The entire data-driven ML technique, free from observers’ or experts’ opinions, is advantageous and meanwhile disadvantageous. The analysis may avoid human bias or observer bias. However, it was the nature of our database that yielded such results. The results of a completely data-driven study, such as this study, may not be applicable to other populations and further external validation may be needed. It is unlikely to utilize a single method to fit all circumstances. The choice of algorithms or models may depend on the nature of a particular dataset (population with different clinical characteristics) or the goals of a desired model. Still, we provided a potential methodology for constructing predictive models by using ML techniques.

This study also has limitations. First, our study was a retrospective study, and data of long-term follow up are not included in the TNN database. We only collected data from Taiwan and thus data relating to other populations were not available. Due to confidentiality of patient information and the policy of data collection from TNN, the GA was presented only in completed weeks and the BW was presented only in 100 g bins. Therefore, the z scores could not be calculated. We used IBM SPSS Statistics 25 to examine the collinearity between GA and BW. The variance inflation factors were examined and indicated no significant collinearity that needs to be corrected. However, when we performed collinearity diagnosis, the condition index showed moderate collinearity. This different results might be due to the nature of our database, that we only have complete GA and categorized BW (per 100 g). This is a limitation of our database. Despite the fact that BW and GA may have mild to moderate collinearity, the software-based attribute selection by Weka did not exclude any of the two variables. Moreover, BW and GA were a well-known important risk factor when predicting BPD. We therefore did not exclude any of the two variables in our models. In addition, we excluded infants who were discharged before 36 weeks’ PMA because we did not have the respiratory support data at discharge for infants from before 2018. In Taiwan, our clinical experience suggests that an infant who can be discharged before 36 weeks’ PMA is more likely to not have BPD or have BPD of lower severity (e.g., mild BPD). Thus, the severity of BPD may have been overestimated in our cohort. We predicted BPD in infants’ early life at the outset. However, some of the risk factors that are reportedly associated with BPD were not included in our study, such as a maternal history of smoking or the presentation or treatment of patent ductus arteriosus or severe intraventricular hemorrhage^34–36^. Finally, external validation was not performed in our study.

The predictive models developed in our study exhibited promising performance and external validation is necessary in the future. Moreover, the association between our predictions and the infants’ actual long-term outcomes must also be evaluated and discussed in the future.

## Conclusions

This study developed prediction models to predict the probability of death or respiratory support at 36 week’s PMA from a preterm infant’s early-life characteristics. The logistic regression algorithm yielded the optimal performance among all the algorithms. Estimators were developed for use in clinical applications after the models were simplified through attribute selection.

## Supplementary Information

Supplementary Information 1.

Supplementary Information 2.

## Data Availability

According to the Taiwan Neonatal Network (TNN) Database Availability and Application Policy, although being anonymized and de-identified, the data are confidential. The data from TNN must only be available to individuals who have access for the authorized research. The data from this study are available from the corresponding author upon reasonable request.
