# Supplementary material for: Machine learning to predict late respiratory support in preterm infants: a retrospective cohort study
Source: Sci Rep. 2023 Feb 17;13:2839. doi: 10.1038/s41598-023-29708-4 (PMC9938227; doi:10.1038/s41598-023-29708-4)
Supplement: Supplementary file 2 — Supplementary Information 2. [file 41598_2023_29708_MOESM2_ESM.docx]

**Machine Learning to Predict Late Respiratory Support in Preterm Infants: A Retrospective Cohort Study**

Tsung-Yu Wu^1,2^, Wei-Ting Lin^2^, Yen-Ju Chen^2^, Yu-Shan Chang^2^, Chyi-Her Lin^2,3,4^ & *Yuh-Jyh Lin^2^

^1^Department of Pediatrics, Ditmanson Medical Foundation Chia-Yi Christian Hospital, Chia-Yi, Taiwan; and ^2^Department of Pediatrics, National Cheng Kung University Hospital, College of Medicine, National Cheng-Kung University, Tainan, Taiwan; ^3^Department of Pediatrics, E-Da Hospital, I-Shou University, Kaohsiung, Taiwan; and ^4^School of Medicine for International Students, College of Medicine, I-Shou University, Kaohsiung, Taiwan

**Supplementary Materials**

**Supplementary Excel File. Outcome Estimator**

We used Orange to calculate the intercept and coefficient of each selected attribute for the prediction models constructed using logistic regression. Equations were developed and outcome estimators for clinical applications were established using Microsoft Excel 2016.­­­ By entering the early-life characteristics of a premature baby, we could calculate the possibility of the various outcomes readily for clinical application.
